# The effect of early burn injury on sensitivity to future painful stimuli in dairy heifers

**DOI:** 10.1371/journal.pone.0233711

**Published:** 2020-06-03

**Authors:** Sarah J. J. Adcock, Cassandra B. Tucker

**Affiliations:** 1 Center for Animal Welfare, Department of Animal Science, University of California, Davis, California, United States of America; 2 Animal Behavior Graduate Group, University of California, Davis, California, United States of America; University of Palermo, ITALY

## Abstract

Animals that experience painful procedures as neonates are more sensitive to pain later in life. We evaluated whether disbudding with a heated iron at 3 (n = 12), 35 (n = 9), or 56 (n = 20) d of age affected heifers’ pain responses to vaccine injections at 11 mo of age. Heifers responded to the injection procedure with struggling and changes in eye temperature and heart rate variability compared to a sham procedure the day before, and still had a heightened response 6 d later, regardless of disbudding age. However, some heart rate variability indices suggested increased sympathetic dominance in heifers disbudded at 35 d, compared to the other 2 age groups, independent of the injection procedure. We also found that heifers disbudded at 3 or 35 d had a higher mean heart rate after the injection procedure compared to those disbudded at 56 d. We conclude that: (1) heifers find injections aversive; and (2) there is some evidence that disbudding age influences autonomic nervous system activity later in life.

## Introduction

Performing painful procedures near birth can cause lasting changes in pain perception, extending into adulthood [1–3]. In rats for instance, neonatal painful procedures including hindpaw incision [4], colon irritation [5], or a stimulated peripheral inflammation with complete Freund's adjuvant [6] or carrageenan [7] all cause hypersensitivity to further painful stimuli as adults. Insults that occur after the first postnatal week in rats do not have this lasting effect [8]. Similarly, human infants who experienced surgery [9] or suffered burn injuries [10] were more sensitive to noxious stimulation later in childhood or adolescence. Although most studies have focused on humans and rodents, there is also evidence for long-term effects of neonatal pain in precocial species [11, 12]. For example, ewes that undergo a painful procedure, tail-docking [13], at 3- or 4-d of age showed more pain responses during parturition as adults than undocked ewes [12].

The mechanisms underlying persistent alterations in pain processing after neonatal injury remain unclear, but likely involve changes in the interdependent nervous, endocrine, and immune systems [14]. These 3 systems undergo significant changes in the neonatal period, during which time stressors, such as tissue injury or a noxious stimulus, can influence subsequent development [15, 16]. Due to the reciprocal communication between these systems, altered programming in any one of them may manifest as changes in pain sensitivity later in life. Indeed, non-injurious early adverse experiences, such as psychological stress [17] or bacterial infection [12, 18, 19], can also alter future responses to pain. These long-term effects may occur via actions on the hypothalamic pituitary adrenal axis, peripheral and central immune system, spinal and supraspinal pathways, and the autonomic nervous system [15].

The long-term effects of early pain may be particularly important for the welfare of agricultural animals, as painful husbandry procedures are often performed at a young age. In dairy heifers for instance, disbudding, a painful procedure to stop horn growth, is performed on 94% of U.S. dairies to prevent horn-related injuries to humans and other animals [20, 21]. A common disbudding practice is to cauterize the horn-growing tissue with a heated iron when heifers are 0 to 8 wk of age. After this age, the horn buds attach to the skull and more invasive and extensive procedures are required for removal in older animals. U.S. veterinary and industry groups recommend that hot-iron disbudding be performed at the youngest practical age, which is increasingly interpreted as the first week of life [22–24]. Although disbudding should unequivocally be done before 8 wk of age to avoid the need for more invasive procedures, we lack empirical evidence about how disbudding at different time points within this window might affect heifer welfare. Given the consequences of early painful experiences in other species, it is possible that disbudding near birth may lead to long-term alterations in pain sensitivity.

Our objective was to determine whether disbudding age affects heifers’ behavioural and physiological responses to a painful husbandry procedure later in life. We quantified pain responses using observations of struggling behaviour recorded concurrently with non-invasive measures of physiological function, specifically heart rate variability and eye temperature. Heart rate variability reflects changes in the balance between the parasympathetic and sympathetic branches of the autonomic nervous system; whereas eye temperature is thought to be regulated by activity of the sympathetic nervous system and hypothalamic pituitary adrenal axis [25]. Changes in both heart rate variability and eye temperature have been observed after painful procedures in cattle [26, 27]. We predicted that heifers disbudded at 3 d of age would show a heightened pain response to vaccine injections at 11 mo compared to those disbudded at 35 or 56 d, the latter age being the upper limit recommended for preventing horn growth by cauterization.

## Methods

This study was conducted from June to September 2017 at the University of California Davis Dairy Facility. All experimental protocols were approved by and carried out in accordance with the University of California Davis Institutional Animal Care and Use Committee (protocol #19842).

### Animals and housing

We used 8 Jerseys and 33 Holsteins (mean ± SD age at injection: 338 ± 7 d) born at the UC Davis dairy between June 20 and September 28, 2016. These heifers were previously enrolled in an experiment to evaluate wound healing and sensitivity after disbudding at different ages [28]. In this previous study, the animals were disbudded at 3–4 d of age (n = 12), 34–35 d of age (n = 9), or 56 d of age (n = 20). All heifers received pain relief at the time of disbudding as to do otherwise would be unethical, considering that the procedure is unequivocally painful [21]. Briefly, heifers were given a cornual nerve block with 5 mL unbuffered 2% lidocaine hydrochloride before a heated iron (X50, Rhinehart Development Corp., Spencerville, IN) was applied to each horn bud for approximately 15–20 s. The iron was fitted with a 1.3 cm tip and heated between 400–500°C. We did not remove the horn bud (bud-in approach). Heifers received 1 mg/kg oral meloxicam immediately after disbudding. There is good evidence that pain persists in the weeks after disbudding [28–30], long after the drugs’ analgesic effects have dissipated.

Heifers were reared according to the facility’s standard operating procedure, and none were removed from the herd between the time of disbudding and the trial. At 10 mo of age, heifers were moved to a 16 x 30 m pen that housed20-25 individuals between 10 and 12 mo of age. At any one time, the pen included heifers from all 3 disbudding age groups, as well as heifers not included in the experiment. The pen included a roofed area with a 15-cm deep rice hull pack. Heifers were fed a dry total mixed ration once daily. Water was available ad libitum. All heifers remained in the herd following data collection.

### Treatments

We assigned heifers to cohorts based on birth order (4–5 heifers/cohort, 9 cohorts total). Disbudding ages were balanced as evenly as possible across cohorts, with at least 2 ages represented in each. We observed cohorts in 3 trials: control (C-1), injection the next day (I0), and a control 6 d later (C6).

On trial days, the cohort was separated from the rest of their penmates using a gate that divided the pen in half. The cohort was then restrained in a headlock (S1 Fig), with an empty gate between each heifer. There were two pens on either side of the headlock that allowed visual, but not physical, access to other heifers. We observed one individual at a time, and order was balanced by disbudding age. Each heifer was provided ad libitum access to a total mixed ration in a bucket. We removed the bucket 2 min before data collection and replaced it at the end of the 10-min sampling period for each heifer. Data were collected for 5 min before and after the injection. The injections consisted of 2 mL of IBR-BVD-BRSV-PI3-Lepto 5 vaccine (Bovi-Shield Gold FP5 L5) and 2 mL of a leptospirosis vaccine (Spirovac) administered subcutaneously on the right and left side of the neck, respectively. These vaccinations were part of the facility’s SOP and no drugs were given for the sole purpose of the study. The Bovi-Shield vaccine was always given first and a fresh 18 gauge 1.9 cm needle was used for each injection. In control trials, an empty needleless syringe was depressed against each side of the neck. At the time of the procedure, the same person (SJJA) approached the heifer from the front and reached an arm through the headlock to administer each injection. The injection procedure took 30 ± 12 s (mean ± SD). Trials occurred between 0700 and 1100 h. Heifers were restrained for 1 to 1.5 h in the headlock. The day before C-1, heifers were habituated to restraint in the headlock and to wearing the heart rate equipment over 1 h. At the beginning of the habituation period, each heifer was clipped down the left side of the thorax before being fitted with the heart rate belt for 10 min.

### Behaviour

We positioned 2 cameras (HC-V180, Panasonic, Japan) on tripods 3 m in front of and 5 m behind the heifer. The video was analysed for 60 s after the first injection for the frequency of 6 behaviours (Table 1). A single observer, blind to treatment, scored all video using BORIS (Behavioral Observation Research Interactive Software) [31]. All behaviours had good intra-observer reliability (25% of data used for calculation; Intraclass correlation coefficient > 0.80).

**Table 1:** Behavioural definitions used to evaluate heifers’ responses after receiving an injection or sham procedure while restrained in a headlock.

Behaviour	Definition	Camera angle
Pulling back	Moving the body such that the headlock comes in contact with the ears and/or cheekbones of the heifer. Each time the heifer loses contact with the bar counts as a separate bout.	Front
Thrusting forward	Movement of the heifer’s body in a straight-on motion such that the headlock comes into contact with her shoulders. Each time the heifer loses contact with the bar counts as a separate bout.	Front
Chin thrust	Nose of the heifer lifts up showing the underside of the chin/jaw. There must be at least 1 s between chin thrusts to count as a separate bout.	Front
Head shake	The heifer rotates her head at least once to both sides (left and right) in a successive rapid motion. There must be at least 1 s between successive rapid side-to-side movements to count as a new head shake.	Front
Hindleg lift	Either hind leg is raised such that the hoof is no longer in contact with the ground and placed back down.	Back
Tail flick	Tail crosses the midline of either rear leg in an outwards and upwards motion.	Back

### Eye temperature

We used an infrared thermal camera (T430, FLIR Systems, Inc., Wilsonville, OR) to photograph the left eye at a distance of 0.5 m. One photo was taken every 30 s beginning 5 min before and ending 5 min after the first injection. A single observer recorded the maximum temperature (°C) of the medial posterior palpebral border of the lower eyelid and the lacrimal caruncle using image analysis software (ResearchIR Max, FLIR Systems, Inc.; Intra-observer reliability using 20% of data: Intraclass correlation coefficient = 0.99). Eye temperature data for 3/122 trials were missed due to equipment failure.

### Heart rate variability

We recorded continuous interbeat intervals from 5 min before to 5 min after the first injection with a heart rate monitor (v800, Polar Electro Oy, Helsinki, Finland). The monitor consisted of an electrode belt that transmitted data to the recording watch through Bluetooth wireless technology. Two min before data collection, we applied electrode gel and water to the belt and secured it around the heifer’s thorax with the electrode contact sites over the previously clipped area. Data were downloaded using Polar software (FlowSync, version 2.3.8, Polar Electro Oy, Helsinki Finland). We missed heart rate data for 9/122 trials due to equipment failure.

We used data sets divided into 5-min periods before and after the start of the first injection to fulfill recommendations for heart rate variability analysis [32]. We calculated heart rate variability indices with Kubios HRV 3.0.2 software [33]. Time-domain measures analyzed were mean heart rate and root mean square of successive differences (RMSSD). Frequency domain measures were high frequency power (HF) and the low-frequency to high-frequency ratio (LF/HF). The HF band was defined as 0.20 to 0.58 Hz [32], and HF was reported in normalized units. Nonlinear measures were the SD2/SD1 ratio and sample entropy. Artefacts were corrected using the software’s “very low” threshold. We excluded data sets with ≥ 6% artefacts (21 of 113 pre-injection periods discarded; 14 of 113 post-injection periods discarded).

### Statistical analysis

One Jersey disbudded at 3 d of age escaped from the headlock after receiving the first injection in I0, and therefore is missing post-injection data for that trial. One Holstein disbudded at 56 d of age was not observed in C6 due to an injury unrelated to this experiment.

#### Behaviour

Counts of individual behaviours were summed as a single measure of struggling (for information about each individual behaviour, see S1 Table). We assessed the effect of trial (C-1, I0, C6), disbudding age (3, 35, 56), and their interaction on the frequency of struggling with a mixed-effects negative binomial model.

#### Eye temperature

Based on visual inspection of the data, we calculated means for maximum eye temperature for 3 time intervals relative to the first injection: -5-0 min, 0–1 min, and 2–5 min. We used linear mixed models to test the effect of trial, disbudding age, and their interaction on maximum eye temperature at each of the 3 time intervals. We also tested these variables’ effects on the relative change in eye temperature from the first to the second time interval, and from the second to the third time interval.

#### Heart rate variability

We ran separate linear mixed models for the 5-min periods before and after the first injection for each heart rate variability measure. We included trial, disbudding age, and their interaction as fixed effects. We also tested for these effects on the change in heart rate over a 30-s period after the first injection compared to baseline (30 s before injection) using a linear mixed model.

All analyses were conducted in R, version 3.4.1 [34]. General and generalized linear mixed models were fitted with the “nlme” package [35] and “glmmADMB” package [36], respectively. We confirmed homogeneity and normality of residuals using residuals vs fits plots and qq plots. In all models, heifer was fitted as a random effect. When a main or interaction effect was present (P < 0.10), we calculated pairwise contrasts using Tukey’s method with the “emmeans” package [37].

## Results

### Behaviour

**Fig 1:**
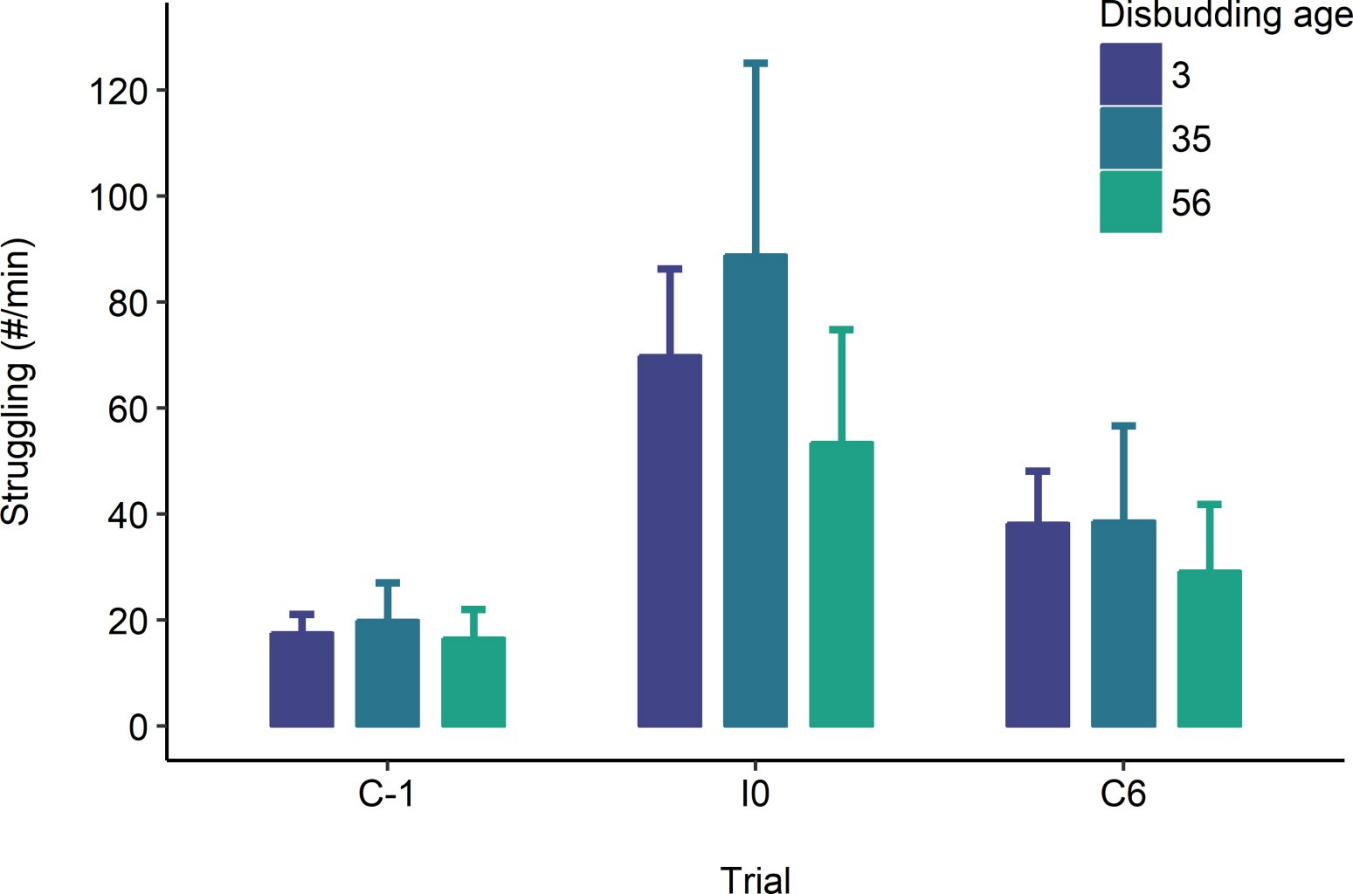
Estimated marginal mean ± SE count of struggling behaviour in 11-mo-old heifers receiving 2 consecutive injections, one on each side of the neck (I0), while restrained in a headlock. Heifers were also observed in a sham procedure the day before (C-1) and 6 d after (C6) the injections. Behaviours were scored for 60 s following the start of the procedure. Heifers were previously disbudded at 3 (n = 12), 35 (n = 9), or 56 (n = 20) d of age. Data are back-transformed from the log scale.

Heifers struggled more in the injection trial (I0) than in the control ones (C-1 and C6), and more struggling occurred in C6 compared to C-1 (Trial: *X*^2^_2_ = 58.79; P < 0.001; Fig 1). We did not observe an effect of disbudding age (*X*^2^_2_ = 0.52; P = 0.773) or an interaction with trial (*X*^2^_4_ = 1.50; P = 0.827). Tail flicks and hindleg lifts were the most frequent struggling behaviours observed (S1 Table).

### Eye temperature

**Fig 2:**
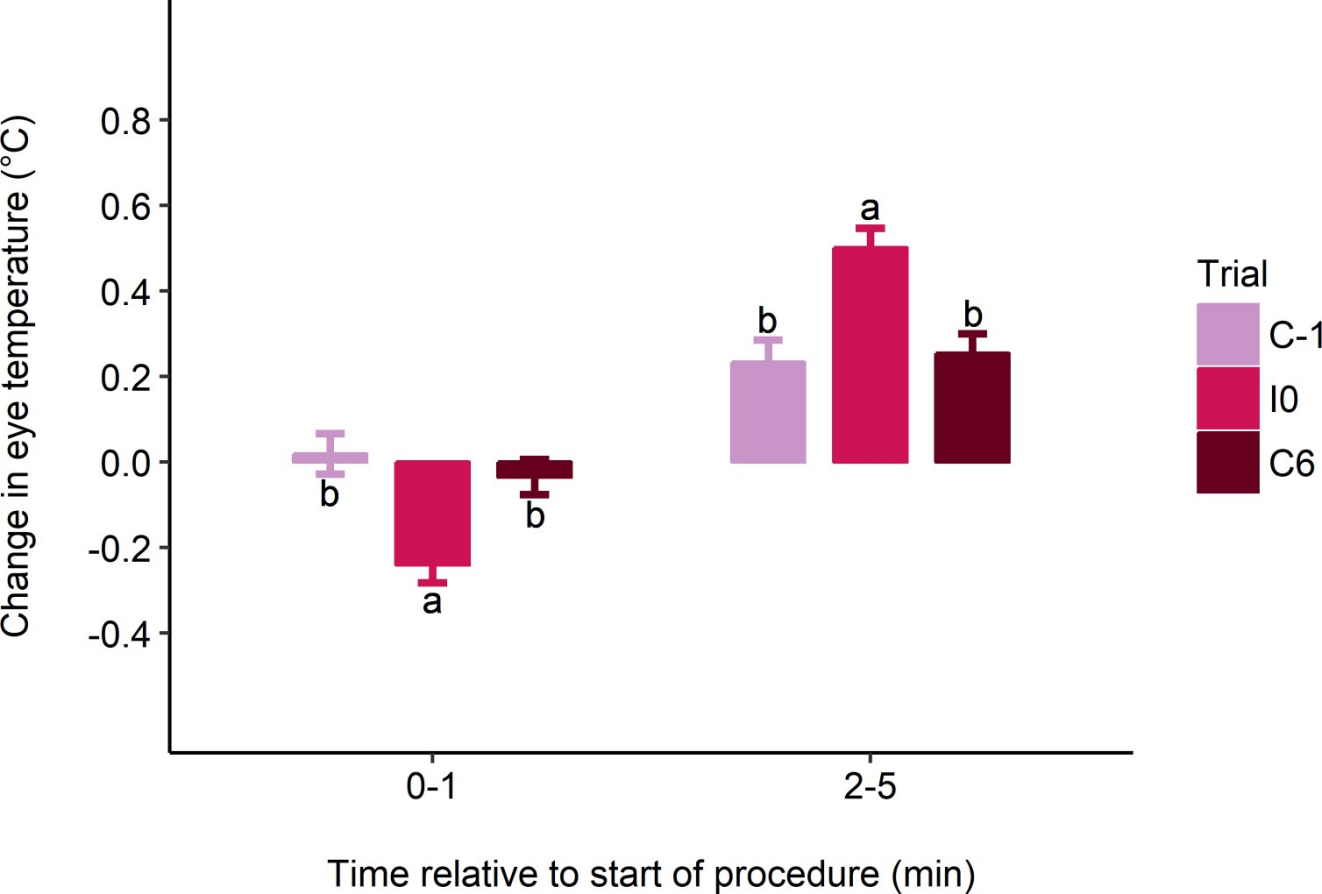
Estimated marginal mean ± SE change in maximum eye temperature (°C) from baseline (-5-0 min) to 0–1 min, and from 0–1 min to 2–5 min post-treatment. Heifers received 2 consecutive injections, one on each side of the neck (I0), as well as a sham procedure the day before (C-1) and 6 d after (C6) the injections. The injection procedure began at 0 min and took approximately 30 s. Different superscripts indicate trial differences (P < 0.05) within time intervals.

**Fig 3:**
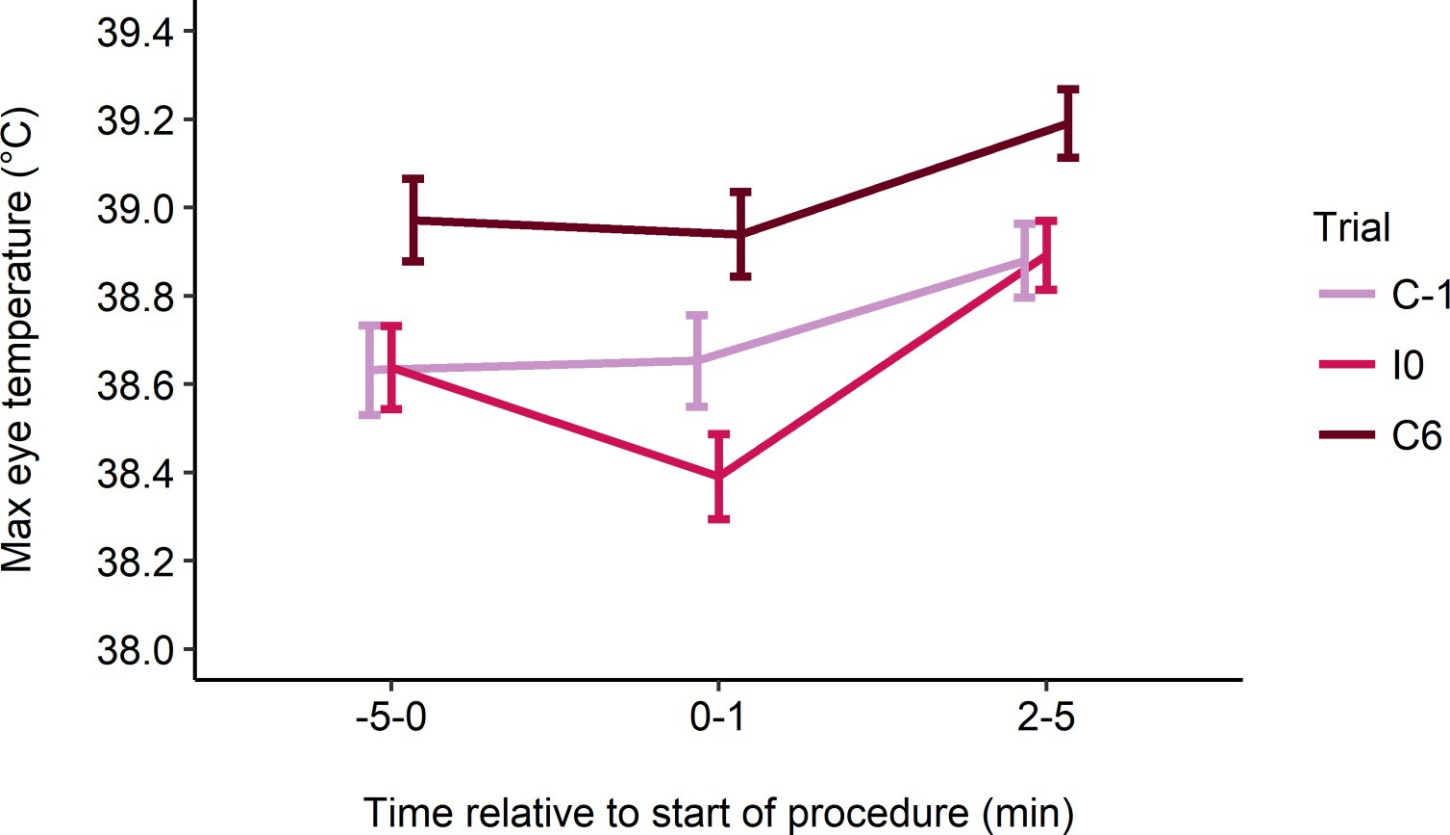
Estimated marginal mean ± SE maximum eye temperature (°C) at baseline (-5-0 min), and 0–1 to 2–5 min post-treatment. Heifers received 2 consecutive injections, one on each side of the neck (I0), as well as a sham procedure the day before (C-1) and 6 d after (C6) the injections. The injection procedure began at 0 min and took approximately 30 s.

Eye temperature decreased from baseline (-5-0 min) in the 1 min following the first injection in I0, but not in control trials (mean ± SE change in eye temperature: C-1: 0.02 ± 0.05°C; I0: -0.24 ± 0.04°C; C6: -0.04 ± 0.04°C; F_2, 64_ = 3.15; P = 0.049; Fig 2). Eye temperature then increased between the second (0–1 min) and third (2–5 min) intervals in all trials, but this increase was greater in I0 than in control trials (C-1: 0.23 ± 0.05°C; I0: 0.50 ± 0.05°C; C6: 0.25 ± 0.05°C; F_2, 64_ = 4.98; P = 0.010; Fig 2). Eye temperature at each time interval was higher in C6 compared to C-1 and I0 (F_2, 64–66_ ≥ 6.10; P < 0.004; Fig 3). Neither disbudding age nor its interaction with trial affected absolute eye temperature at each time interval or its change across intervals.

### Heart rate variability

**Fig 4:**
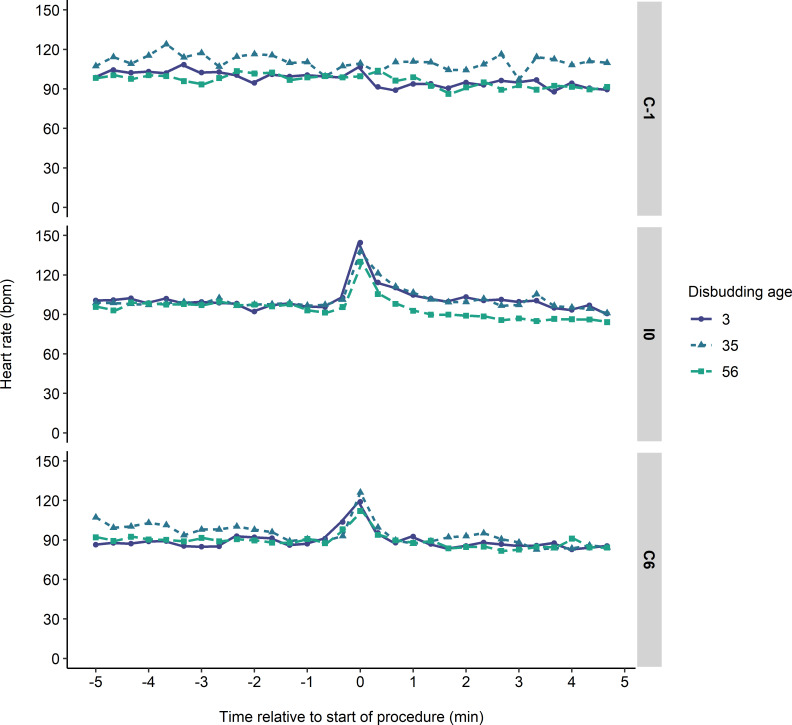
Mean heart rate during the 10-min sampling period for 11-mo-old heifers receiving 2 consecutive injections, one on each side of the neck (I0), while restrained in a headlock. Heifers were also observed in a sham procedure the day before (C-1) and 6 d after (C6) the injections. Heifers were previously disbudded at 3, 35, or 56 d of age. The injection procedure began at 0 min and took approximately 30 s.

Fig 4 shows the heart rate response through time for the different disbudding age groups in each trial. Heart rate was above baseline in the 30 s following the first injection in I0 and C6, but not in C-1 (change in heart rate; C-1: 3 ± 3 bpm; I0: 34 ± 3 bpm; C6: 19 ± 3 bpm; F_2, 66_ = 12.97, P < 0.001). Neither disbudding age nor its interaction with trial affected the change in heart rate following treatment.

#### Pre-injection

Table 2 summarizes the heart rate variability indices for the 5-min period before the first injection. Heifers disbudded at 35 d of age had lower RMSSD than heifers disbudded at 3 d of age (Age: F_2, 37_ = 5.83, P = 0.006). HF was lower in heifers disbudded at 35 d of age than those disbudded at 3 d of age in C-1 (Trial*Age: F_4, 47_ = 2.67, P = 0.044). In C-1 and I0, the LF/HF ratio was higher in heifers disbudded at 35 d of age than those disbudded at 3 or 56 d of age (Trial*Age: F_4, 47_ = 4.06, P = 0.007). We did not observe effects on mean heart rate, SD2/SD1, or sample entropy.

**Table 2:** Heart rate and heart rate variability (HRV) parameters (estimated marginal mean ± SE) in 11-mo-old heifers restrained in a headlock during the 5-min period before the injection procedure.

HRV parameter	Disbudding age	C-1	I0	C6
Heart rate (bpm)	3	90 ± 4	94 ± 4	84 ± 4
	35	97 ± 5	97 ± 4	91 ± 4
	56	90 ± 3	91 ± 3	85 ± 3
RMSSD (ms)	3	81 ± 9	55 ± 10	52 ± 9
	35	28 ± 15	30 ± 11	46 ± 11
	56	48 ± 8	46 ± 7	48 ± 8
HFnorm	3	64 ± 7^a^	58 ± 7	48 ± 7
	35	23 ± 11^b^	33 ± 8	47 ± 8
	56	45 ± 6^ab^	45 ± 5	57 ± 6
LF/HF ratio	3	0.6 ± 1.7^b^	0.7 ± 1.8^b^	3.5 ±1.7
	35	15.3 ± 2.8^aB^	8.9 ± 2.1^aB^	2.0 ± 2.1^A^
	56	2.4 ± 1.4^b^	2.7 ± 1.4^b^	2.1 ± 1.4
SD2/SD1 ratio	3	2.0 ± 0.6	1.9 ± 0.6	2.6 ± 0.6
	35	4.0 ± 0.9	3.9 ± 0.7	3.1 ± 0.7
	56	2.4 ± 0.5	2.7 ± 0.5	2.6 ± 0.5
Sample entropy	3	0.45 ± 0.08	0.37 ± 0.08	0.59 ± 0.08
	35	0.42 ± 0.11	0.44 ± 0.09	0.37 ± 0.09
	56	0.58 ± 0.06	0.46 ± 0.06	0.54 ± 0.06
Sample size	3	10	9	10
	35	4	7	7
	56	15	16	15

The procedure consisted of 2 consecutive injections, one on each side of the neck (I0), or a sham procedure the day before (C-1) and 6 d after (C6) the injections. Heifers were previously disbudded at 3, 35, or 56 d of age. Different lower-case superscripts indicate differences between ages within trial (P < 0.05). Different upper-case superscripts indicate differences between trials within age group. Pairwise contrasts within a trial or age group were only calculated when an interaction effect between trial and disbudding age was observed (P < 0.10). RMSSD = root mean square of successive differences; HF = high frequency; LF/HF = low-frequency to high-frequency ratio.

#### Post-injection

Table 3 summarizes the heart rate variability indices for the 5-min period after the first injection. In I0, mean heart rate was higher in heifers disbudded at 3 or 35 d of age compared to those disbudded at 56 d of age (Trial*Age: F_4, 52_ = 2.17, P = 0.085). Mean heart rate was higher in I0 than in C6 for all disbudding age groups, and was higher than in C-1 for heifers disbudded at 3 d of age. Sample entropy was lower in I0 and C6 compared to C-1 (Trial: F_2, 52_ = 4.24, P = 0.020). In C-1, SD2/SD1 and LF/HF were higher in heifers disbudded at 35 d of age than at 3 or 56 d of age, and then decreased in subsequent trials (Trial*Age: F_4, 52_ ≥ 2.08, P ≤ 0.096). We did not observe effects on HF or RMSSD.

**Table 3:** Heart rate and heart rate variability (HRV) parameters (estimated marginal mean ± SE) in 11-mo-old heifers restrained in a headlock during the 5-min period after the injection procedure.

HRV parameter	Disbudding age	C-1	I0	C6
Heart rate (bpm)	3	85 ± 3^B^	97 ± 3^aA^	85 ± 3^B^
	35	93 ± 4^AB^	100 ± 3^aA^	88 ± 3^B^
	56	85 ± 2^AB^	89 ± 2^bA^	82 ± 2^B^
RMSSD (ms)	3	66 ± 8	61 ± 8	72 ± 8
	35	31 ± 12	61 ± 9	59 ± 8
	56	52 ± 6	64 ± 6	62 ± 6
HFnorm	3	53 ± 7	60 ± 7	59 ± 7
	35	30 ± 11	37 ± 8	44 ± 7
	56	46 ± 6	54 ± 6	54 ± 5
LF/HF ratio	3	5.4 ± 1.7^b^	2.2 ± 1.8	0.8 ± 1.7
	35	13.6 ± 2.7^aA^	2.9 ± 2.0^B^	3.0 ± 1.8^B^
	56	2.3 ± 1.4^b^	1.6 ± 1.4	1.2 ± 1.3
SD2/SD1 ratio	3	2.7± 0.5^b^	2.9 ± 0.5	2.3 ± 0.5
	35	6.1 ± 0.8^aA^	3.2 ± 0.6^B^	3.1 ± 0.5^B^
	56	2.7 ± 0.4^b^	2.8 ± 0.4	2.6 ± 0.4
Sample entropy	3	0.48 ± 0.06	0.28 ± 0.06	0.38 ± 0.06
	35	0.59 ± 0.09	0.25 ± 0.07	0.37 ± 0.07
	56	0.55 ± 0.05	0.33 ± 0.05	0.34 ± 0.05
Sample size	3	10	9	10
	35	4	7	9
	56	16	16	18

The procedure consisted of 2 consecutive injections, one on each side of the neck (I0), or a sham procedure the day before (C-1) and 6 d after (C6) the injections. Heifers were previously disbudded at 3, 35, or 56 d of age. Different lower-case superscripts indicate differences between ages within trial (P < 0.05). Different upper-case superscripts indicate differences between trials within age group. Pairwise contrasts within a trial or age group were only calculated when an interaction effect between trial and disbudding age was observed (P < 0.10). RMSSD = root mean square of successive differences; HF = high frequency; LF/HF = low-frequency to high-frequency ratio.

## Discussion

Heifers struggled four-fold more and had greater cardiac and eye temperature responses when receiving vaccinations compared to a sham procedure, indicating that the injections were painful. The higher heart rate response to the injections in heifers disbudded at 3 or 35 d of age suggests that performing painful procedures in the first 5 wk may lead to increased pain sensitivity later in life. In addition, some heart rate variability indices suggested altered sympathovagal balance in heifers disbudded at 35 d compared to the other 2 age groups, pointing to a possible developmental window in which injury leads to long-term changes in autonomic function.

Eye temperature decreased 0.25°C in the 1 min following the first injection in I0, but not in control trials. Eye temperature then increased above baseline between 2–5 min after the first injection/syringe in all trials, but the greatest increase was seen in I0. Others have found a rapid drop in eye temperature after an aversive handling procedure in cattle [38] and disbudding of heifers without local anesthetic [27]. A longer-term increase in eye temperature has been observed in cattle after catheterization [39], cornual nerve blocks [40], castration [41], and disbudding [27]. The neuroendocrine mechanisms underlying this response, however, are not fully understood. Stewart et al [27] suggest that the initial drop may be due to sympathetically-mediated vasoconstriction; whereas the subsequent increase could be associated with vasodilation mediated by the autonomic nervous system and vasodilators released in response to pain [41].

The magnitudes of our heart rate and heart rate variability measures were comparable to previously reported values in cattle [27, 41, 42]. Heart rate increased 34 bpm above baseline in the 30 s following the first injection, similar to the 35 bpm increase seen after disbudding without anesthesia [27]. Sample entropy and LF/HF were lower in the 5 min after the injections in I0 compared to C-1, indicating more heart rate regularity and parasympathetic activity, respectively. The few studies that have evaluated nonlinear heart rate variability indices in farm animals have also reported lower sample entropy in response to stressors, such as surgical castration [43], heat stress [44, 45], and hemorrhagic shock [46] in pigs. The lower LF/HF after the injections is surprising, as stress is typically associated with higher LF/HF, reflecting stimulation of the sympathetic branch of the autonomic nervous system. Indeed, LF/HF increased above baseline in response to disbudding [27], insect harassment [47], and diarrhoea [47] in heifers, and after calving in cows [48]. In contrast, LF/HF decreased after surgical castration in heifers [41] and in humans watching blood-draw and injection videos [49]. Lower LF/HF may reflect a vasovagal response to stress, in which a transient increase in sympathetic activity is followed by sympathetic withdrawal and an increase in vagal tone [50]. Blood donors experiencing vasovagal symptoms, such as dizziness and weakness, had low LF/HF [51]. It is unclear why the heifers exhibited this response to the injections, but it could be triggered by procedures involving skin puncture, as a vasovagal reaction is thought to have evolved, at least in part, to reduce blood loss following injury [50].

The memory of the injection procedure appears to have persisted for at least 6 d, as suggested by a greater behavioural and cardiac response in C6 compared to C-1. Heifers began struggling at the start of the sham injection in C6, presumably in anticipation of a painful event based on previous experience. Before this study, the heifers’ only experience with injections in the headlock was a brucellosis vaccination given between 137 and 179 d of age, more than 5 mo previously. Thus, a single exposure to injections in the headlock may be sufficient for heifers to learn this association for at least 6 d. In contrast, in a rodent conditioning paradigm, 25 pairings of an auditory cue with a noxious stimulus (i.e., laser) were needed before the tone alone was able to elicit escape behaviour [52]. Others have reported that one exposure to electro-immobilization in sheep [53] or branding in steers [54] did not produce aversive responses to the location where the treatment occurred. Although not recorded in the current study, we observed that heifers were more reluctant to enter the headlock in C6. The elevated eye temperature throughout C6 compared to the two previous trials is also suggestive of an aversion to the location where the injections occurred and not just the simulated procedure. Overall, the heightened response in C6 suggests that the heifers perceived the injections as aversive. Since the heifer did not respond to a person touching a needleless syringe to their neck in C-1, the fear response we observed in I0 and C6 is evidence that the injection per se was painful.

Our results add to a growing body of evidence that cattle find injections aversive [40, 55]. Injections are routinely given to livestock for a variety of purposes, including vaccinations, nerve blocks for husbandry and surgical procedures, medications, and reproductive synchronization programs. Injection pain may be modulated by many variables, such as characteristics of the injection fluid (e.g., pH, volume, viscosity), injection speed, needle gauge, route of administration, and injection site [56]. Animal studies on refinements or alternatives for injection procedures are sparse. Ede et al [55] found that subcutaneous injections were less aversive than the intramuscular route in heifers. Another study in heifers found no benefit of applying a topical anesthetic to the injection site before administering a cornual nerve block [40].

We did not observe an effect of disbudding age on struggling or changes in eye temperature, but there were differences in heart rate responses to injections. Heifers disbudded at 3 or 35 d had a heart rate averaging approximately 10 bpm higher than those disbudded at 56 d in the 5-min period after the injections, suggesting that injury incurred at these ages increased pain sensitivity later in life. In a previous study using the same individuals, we found that the heifers disbudded at 3 d were more sensitive to pressure applied to their rump in the weeks following injury compared to those disbudded at 35 d [28]. Although we interpreted this finding as evidence that disbudding near birth may lead to widespread hyperalgesia, we note that it could alternatively reflect hypoalgesia in the heifers disbudded at 35 d. The apparent discrepancy between our previous study and the current one may be explained by the fact that the mechanisms underlying long-term changes in pain sensitivity likely differ based on the developmental window in which the neonatal insult occurred [57]. Thus, it is possible that disbudding at 3 vs 35 d results in different trajectories of neuro-endocrine-immune changes, that eventually converge on the same increased pain sensitivity in adolescence. Furthermore, in some cases mechanical hypersensitivity associated with early injury only emerges later in life [58], and could explain why it was not observed in the heifers disbudded at 35 d in our previous study.

Consistent with the idea that disbudding at 3 vs 35 d may differentially affect physiological functions, heifers disbudded at 35 d had lower parasympathetic indices of heart rate variability (RMSSD, HF) and higher sympatho-vagal indices (LF/HF, SD2/SD1) than the other 2 age groups, independent of the injection procedure. This pattern may reflect decreased vagal tone, increased sympathetic drive, or both [59]. Long-term alterations in autonomic activity consistent with increased arousal have been observed after early painful experiences in humans [60, 61] and after a neonatal immune challenge in rats [62]. Autonomic imbalance is linked to many pathologies, including cardiovascular disease [63], chronic pain [64], depression [65], and all-cause mortality [63]. Given the implications an automatic imbalance could have for cattle welfare, longer-term studies are needed to determine the consistency and magnitude of a disbudding age effect on autonomic activity.

It is unclear why disbudding at 35 d, but not at 3 or 56 d, would lead to altered autonomic activity, but a possible explanation may lie in the transition from passive to active immunity. Levels of maternal antibodies gained through passive transfer peak a few days after birth [66] and may protect the calf against inflammatory stressors such as disbudding. Maternal antibodies decrease around 3 to 4 wk of age, when the calf is not yet producing enough of its own antibodies to compensate for this depletion, resulting in an increased susceptibility to infection during this period [66, 67]. Endogenous production of antibodies rises in the following weeks, and by weaning calves have a more developed active immunity. Interestingly, rats that were challenged with a bacterial infection at 14 or 21 d, but not at 7 or 28 d, had attenuated fever responses to adult infection [68]. This window coincides with a rapid depletion of maternal antibodies in the rat, and low levels of endogenous antibodies [69]. Thus, we speculate that exposure to an inflammatory insult during this “window of susceptibility”–when animals are no longer protected by passive immunity and active immunity has not been stimulated [67]–may influence physiological development, and that further investigation is warranted.

### Limitations

Our small and variable sample size indicates that caution is warranted over the disbudding age effects observed in the HRV data. It is possible that the differences we observed in autonomic activity in calves disbudded at 35 d compared to 3 or 56 d are artefacts of the low sample size in this group, as well as the large number of HRV measures, which increases the Type I error rate. Thus, these results should be considered exploratory, and further research is needed to better understand the implications of disbudding at different ages.

Disbudding is only one of several experiences that could be considered adverse in a heifer’s early rearing environment. In addition to experiencing other painful procedures, such as ear tagging and vaccinations, calves in the U.S. are typically removed from the mother immediately after birth and reared in individual hutches [70]. Rodent models indicate that maternal deprivation and social isolation adversely affect brain development and behaviour [71]. It is possible that these additional stressors led to long-term physiological and behavioural alterations, potentially dampening a disbudding age effect on pain sensitivity.

Another limitation of the current study was that all animals were disbudded, and we cannot rule out that some degree of altered pain sensitivity occurred in all 3 age groups. Unfortunately, the inclusion of a non-disbudded control group was not possible as modern dairy farms are not perceived as safe environments for raising horned animals. Polled (i.e., naturally hornless) heifers could serve as an alternative control, but they are still rare in the dairy industry, including at our facility.

## Conclusion

Overall, our results provide some evidence that a painful procedure in early life may cause long-lasting changes in autonomic activity, but whether it specifically affects reactivity to future painful stimuli or basal activity may depend on the developmental window when tissue damage occurred. A better understanding of postnatal sensitive periods in heifers is needed before recommendations can be made about the best age to perform disbudding. As a long-term solution, breeding polled animals will eliminate the need for this painful procedure.

## Supporting information

S1 Fig A Jersey heifer restrained in the headlock. (JPG)

S1 Table Behavioural counts (mean ± SE) in heifers receiving 2 consecutive injections on each side of the neck (I0) while restrained in a headlock. Heifers were also observed in a sham procedure the day before (C-1) and 6 d after (C6) the injections. Behaviours were scored for 60 s following the start of the procedure.
(DOCX)
