# Supplementary figures and images for: The effect of early burn injury on sensitivity to future painful stimuli in dairy heifers
Source: PLoS One. 2020 Jun 3;15(6):e0233711. doi: 10.1371/journal.pone.0233711 (PMC7269268; doi:10.1371/journal.pone.0233711)

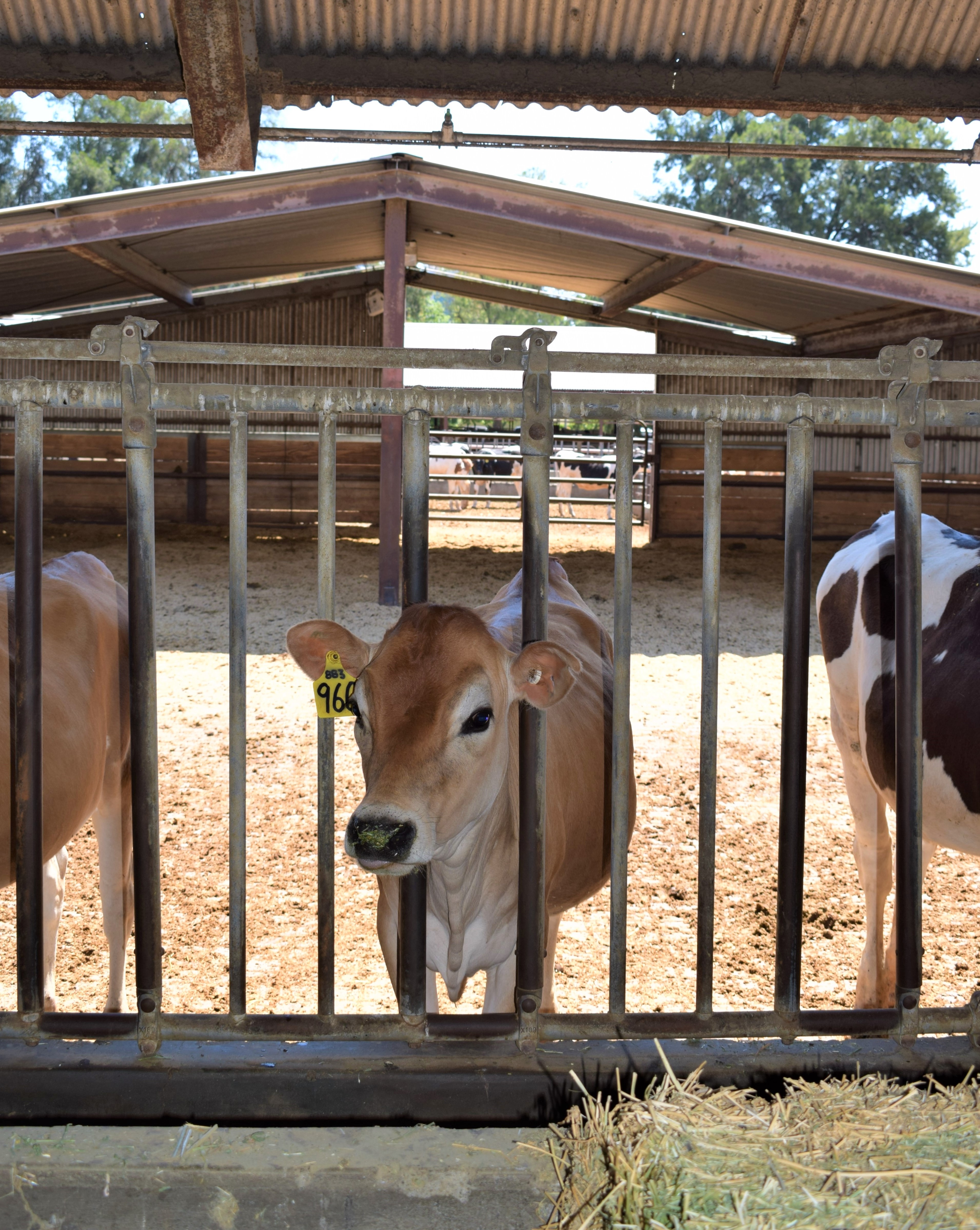

Supplement: S1 Fig — (JPG) [file pone.0233711.s001.jpg]
