# Supplementary material for: The effect of early burn injury on sensitivity to future painful stimuli in dairy heifers
Source: PLoS One. 2020 Jun 3;15(6):e0233711. doi: 10.1371/journal.pone.0233711 (PMC7269268; doi:10.1371/journal.pone.0233711)
Supplement: S1 Table — Heifers were also observed in a sham procedure the day before (C-1) and 6 d after (C6) the injections. Behaviours were scored for 60 s following the start of the procedure. (DOCX) [file pone.0233711.s002.docx]

**S1 Table.** **Behavioural counts (mean ± SE)** **in heifers receiving 2 consecutive injections on each side of the neck (I0) while restrained in a headlock.**

| **Behaviour** | **C-1** | **I0** | **C6** |
| --- | --- | --- | --- |
| Pulling back | 1 ± 0 | 3 ± 0 | 2 ± 0 |
| Thrusting forward | 1 ± 0 | 4 ± 0 | 1 ± 0 |
| Chin thrust | 1 ± 0 | 2 ± 0 | 1 ± 0 |
| Head shake | 0 ± 0 | 3 ± 1 | 1 ± 0 |
| Hindleg lift | 2 ± 0 | 15 ± 1 | 6 ± 1 |
| Tail flick | 14 ± 2 | 45 ± 4 | 26 ± 3 |

Heifers were also observed in a sham procedure the day before (C-1) and 6 d after (C6) the injections. Behaviours were scored for 60 s following the start of the procedure.


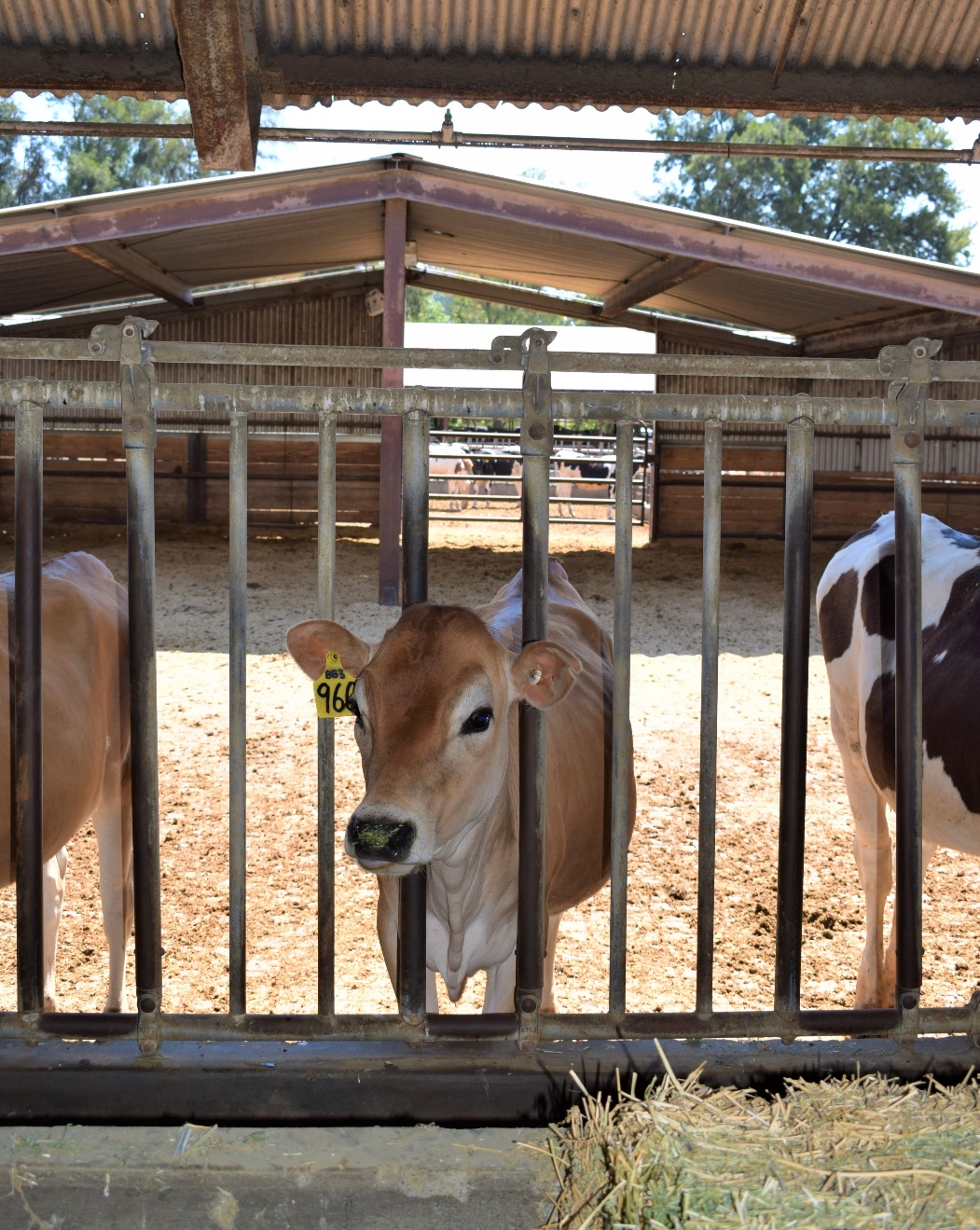


**S1 Fig.** **A Jersey heifer restrained in the headlock.**
